# Checklist of the families Opetiidae and Platypezidae (Diptera) of Finland

**DOI:** 10.3897/zookeys.441.7639

**Published:** 2014-09-19

**Authors:** Gunilla Ståhls

**Affiliations:** 1 Finnish Museum of Natural History, Zoology Unit, P.O. Box 17, FI-00014 University of Helsinki, Finland

**Keywords:** Checklist, Finland, Diptera, Opetiidae, Platypezidae

## Abstract

A checklist of the Opetiidae and Platypezidae (Diptera) recorded from Finland.

## Introduction

Opetiidae and Platypezidae are small families of small-sized flies. Platypezidae are principally forest insects, and all known larvae develop in fungi. New host fungi records were recently reported in Reemer et al. (2014), Ståhls et al. (submitted) and Ståhls and Rättel (2013).

The Finnish species were last listed by Hackman (1980) and Chandler (2001). Chandler (2001) included one and 21 species of Opetiidae and Platypezidae, respectively. Ståhls and Kahanpää (2006), Winqvist (2011), Ståhls et al. (2014), Reemer et al. (2014) and Ståhls and Rättel (2014) added altogether 12 species of family Platypezidae. *Bolopus furcatus* was included in Hackman (1980) but not in Chandler (2001), Ståhls et al. (2012) reported the taxon from Finland again. *Agathomyia* (subfamily Callomyiinae) is the largest genus of the Platypezidae. A recent study reinstated two *Agathomyia* species from synonymy (*Agathomyia boreella* Zetterstedt and *Agathomyia scutellaris* Zetterstedt) and two species were described as new to science (Ståhls et al. submitted). The taxonomic status of *Agathomyia dahlbomi* (Zetterstedt), presently listed as synonym of *Agathomyia elegantula* (Zetterstedt), remains unclear. *Callomyia elegans* and *Protoclythia modesta* are only known from previous single records, their occurrence has not been verified with new findings.

Chandler and Shatalkin (1998) and Chandler (2001) provided keys to subfamilies, genera and species of adult Palaearctic Platypezidae, and key to genera of known early stages of Platypezidae. Ståhls et al. (submitted) provided a key to Northern European *Agathomyia* spp.

**Number of species (Opetiidae, Platypezidae):**

World: 102, 252 species (Pape et al. 2011)

Europe: 1, 46 species

Finland: 1, 38 species

Faunistic knowledge level in Finland: average

## Checklist

suborder Brachycera Macquart, 1834

clade Eremoneura Lameere, 1906

clade Cyclorrhapha Brauer, 1863

infraorder Aschiza Becher, 1882

superfamily Phoroidea Curtis, 1833

**OPETIIDAE** Rondani, 1856

***OPETIA*** Meigen, 1830

*Opetia nigra* Meigen, 1830

**PLATYPEZIDAE** Fallén, 1815

CALLOMYIINAE Rondani, 1841

***AGATHOMYIA*** Verrall, 1901

*Agathomyia alneti* Ståhls & Rättel, in prep.

*Agathomyia antennata* (Zetterstedt, 1819)

*Agathomyia boreella* (Zetterstedt, 1838)

*Agathomyia cinerea* (Zetterstedt, 1852)

*Agathomyia elegantula* (Fallén, 1815)

= *dahlbomi* (Zetterstedt, 1838)

*Agathomyia falleni* (Zetterstedt, 1819)

*Agathomyia lundbecki* Chandler in Shatalkin, 1985

*Agathomyia sexmaculata* (von Roser, 1840)

*Agathomyia scutellaris* (Zetterstedt, 1838)

*Agathomyia shatalkini* Ståhls, in prep.

*Agathomyia unicolor* Oldenberg, 1928

*Agathomyia vernalis* Shatalkin, 1981

*Agathomyia viduella* (Zetterstedt, 1838)

*Agathomyia wankowiczii* (Schnabl, 1884)

*Agathomyia woodella* Chandler in Shatalkin, 1985

= *elegantula* auct. nec (Fallén, 1815)

*Agathomyia zetterstedti* (Wahlberg in Zetterstedt, 1844)

***CALLOMYIA*** Meigen, 1804

*Callomyia amoena* Meigen, 1824

*Callomyia elegans* Meigen, 1804

= *leptiformis* (Fallén, 1810)

*Callomyia krivosheinae* Shatalkin, 1982

*Callomyia speciosa* Meigen, 1824

= *humeralis* Loew, 1869

MICROSANIINAE Enderlein, 1936

***MICROSANIA*** Zetterstedt, 1837

*Microsania capnophila* Shatalkin, 1985

*Microsania collarti* Chandler, 2001

*Microsania pallipes* (Meigen, 1830)

*Microsania pectipennis* (Meigen, 1830)

= *pectinipennis* emend.

*Microsania straeleni* Collart, 1954

*Microsania vrydaghi* Collart, 1954

PLATYPEZINAE Fallén, 1815

***BOLOPUS*** Enderlein, 1932

*Bolopus furcatus* (Fallén, 1826)

***PARAPLATYPEZA*** Kessel & Maggioncalda, 1968

*Paraplatypeza atra* (Meigen, 1804)

*Paraplatypeza bicincta* (Szilády, 1941)

***PLATYPEZA*** Meigen, 1803

*Platypeza aterrima* Walker, 1836

*Platypeza consobrina* Zetterstedt, 1844

*Platypeza fasciata* Meigen, 1804

*Platypeza hirticeps* Verrall, 1901

***PLATYPEZINA*** Wahlgren, 1910

*Platypezina connexa* (Bohemann, 1858)

***POLYPORIVORA*** Kessel & Maggioncalda, 1968

*Polyporivora boletina* (Fallén, 1815)

*Polyporivora ornata* (Meigen, 1838)

= *infumata* (Haliday, 1838)

*Polyporivora picta* (Meigen, 1830)

***PROTOCLYTHIA*** Kessel, 1950

*Protoclythia modesta* (Zetterstedt, 1844)

***SERI*** Kessel & Maggioncalda, 1966

*Seri obscuripennis* (Oldenberg, 1916)
